# Updates on Robotic CME for Right Colon Cancer: A Qualitative Systematic Review

**DOI:** 10.3390/jpm11060550

**Published:** 2021-06-12

**Authors:** Wanda Petz, Simona Borin, Uberto Fumagalli Romario

**Affiliations:** Division of Digestive Surgery, IEO European Institute of Oncology IRCCS, 20141 Milano, Italy; simona.borin@ieo.it (S.B.); uberto.fumagalliromario@ieo.it (U.F.R.)

**Keywords:** complete mesocolic excision, robotic surgery, right colectomy

## Abstract

Background. Complete mesocolic excision (CME) is a surgical technique introduced with the aim of ameliorating the oncologic results of colectomy. Various experiences have demonstrated favorable oncologic results of CME in comparison with standard colectomy, in which the principles of CME are not respected. The majority of the literature refers to open or laparoscopic CME. This review analyses current evidence regarding robotic CME for right colectomy. Methods. An extensive Medline (Pub Med) search for relevant case series, restricted to papers published in English, was performed, censoring video vignettes and case reports. Results. Fourteen studies (ten retrospective, four comparative series of robotic versus laparoscopic CME) were included, with patient numbers ranging from 20 to 202. Four different approaches to CME are described, which also depend on the robotic platform utilized. Intraoperative and early clinical results were good, with a low conversion and anastomotic leak rate and a majority of Clavien–Dindo complications being Grades I and II. Oncologic adequacy of the surgical specimens was found to be good, although a homogeneous histopathologic evaluation was not provided. Conclusions. Further large studies are warranted to define long-term oncologic results of robotic right colectomy with CME and its eventual benefits in comparison to laparoscopy.

## 1. Background

Complete mesocolic excision (CME) with central vessel ligation (CVL) is a surgical technique first described by West [1] in 2008 and Hohenberger [2] in 2009.

By analogy with the concept of total mesorectal excision (TME) for rectal cancer, and with the aim of improving the radicality of surgery and, therefore, of ameliorating the oncologic results of colectomy for cancer, it involves the complete removal of an intact mesocolic envelope surrounding the colon.

In addition, the technique implies ligature of the supplying vessels at their origin from mesenteric artery and vein, and an extended lymphadenectomy, superimposable to the D3 dissection described by Japanese authors [3].

The surgical community has shown a growing interest in this more radical approach to colon cancer, suggesting that it could play a role in ameliorating the oncologic results of right colectomy [4,5,6,7,8,9,10,11,12,13,14]; however, large randomized clinical trials providing a high level of evidence are lacking.

Therefore, the guidelines of the major international scientific societies still do not mention the need to perform a CME when approaching a colonic cancer [15,16], while the same guidelines make TME for rectal cancer mandatory [17,18].

Regarding the surgical approach to CME, the techniques most used are the open and the laparoscopic techniques, although the latter has been recognized as being technically more challenging, especially with regard to vascular dissection [19].

In this paper, we shall focus on the robotic approach to CME for surgery of right colon cancer, analyzing current evidence and providing a summary of its eventual benefits.

## 2. Methods

The Preferred Reporting Outcomes for Systematic Reviews and Meta-Analyses (PRISMA) guidelines were followed [20] and an extensive Medline (Pub Med) search for relevant case series was performed. The search strategy included “complete mesocolic excision” AND “robotic” AND “right colon” OR “right colectomy” OR “right hemicolectomy”.

Inclusion criteria were English language and detailed description of the surgical technique, including all the particular technical aspects of CME with CVL. Exclusion criteria were case reports, video vignettes and case series in which the CME technique was employed for colonic resections different from right or extended right colectomy; if a single centre published more than a paper, only the one including the greater number of patients was included.

The quality of each included study was assessed using the Newcastle–Ottawa Scale (NOS) [21] and the risk of bias was considered high if NOS total score was <7 or low if NOS total score was 7 or more.

A single reviewer (WP) screened each record retrieved and collected data from each report. Outcomes for which data were sought were: number of patients included in the study, type of robotic platform utilized, details of surgical technique (patient and trocar position, sequence of surgical steps, technique of anastomosis), conversion rate, operative time, intra and postoperative complications, number of retrieved lymph nodes, overall and disease-free survival.

Continuous data are presented as median and range, while categorical data are presented as percentages.

## 3. Results

After duplicate censoring, fourteen studies were included in this review [22,23,24,25,26,27,28,29,30,31,32,33,34,35], and the publication dates ranged from 2013 to 2021.

There are ten retrospective non comparative case series [22,23,27,28,29,30,31,33,34,35] and four comparative studies (three retrospective and one prospective) of robotic versus laparoscopic CME for right colectomy [24,25,26,32]; the patient number ranges from 20 to 202.

Characteristics and principal results of the included studies are reported in Table 1.

According to the NOS score obtained, the risk of bias was high in 4 studies and low in the remaining 10 (Table 2).

## 4. Technical Considerations

The da Vinci Xi robotic platform has been utilized in nine of the fourteen studies [23,25,28,30,31,33,35], the Si platform in two [22,24], the X platform in one [35] and both the Si and the Xi in the remaining two [29,32].

Four different approaches to colonic dissection, CME and lymphadenectomy are reported, the difference essentially residing in the sequence of surgical steps and the direction of detachment of the mesocolon from the retroperitoneum: a “medial-to-lateral” approach [22,24,30,32], a “bottom-to-up” approach [23,25,28,29,31,35], a “top-to-down” approach [26] and a “superior mesenteric vein first” approach [27,33,34].

Patient position on the operative table is similar for the “medial-to-lateral”, the “bottom-to-up” and the “superior mesenteric vein first” approaches: the patient is supine and the operative table is in a slight Trendelenburg position and rotated to the left, in order to expose the surgical field by moving the small bowel in the left abdominal quadrants.

In the “top-to-down” approach, conversely, the patient is in a 30° reverse Trendelenburg position in the first phase of surgery, to facilitate entrance in the lesser sac through the gastrocolic ligament; a different docking is performed in the second part of the procedure.

**Figure 1:**
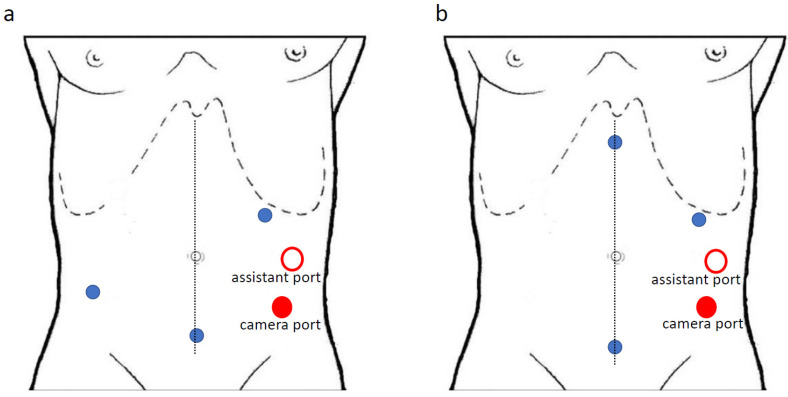
(**a**,**b**): trocars position for “medial-to-lateral” approach with the da Vinci Si^®^ system.

**Figure 2:**
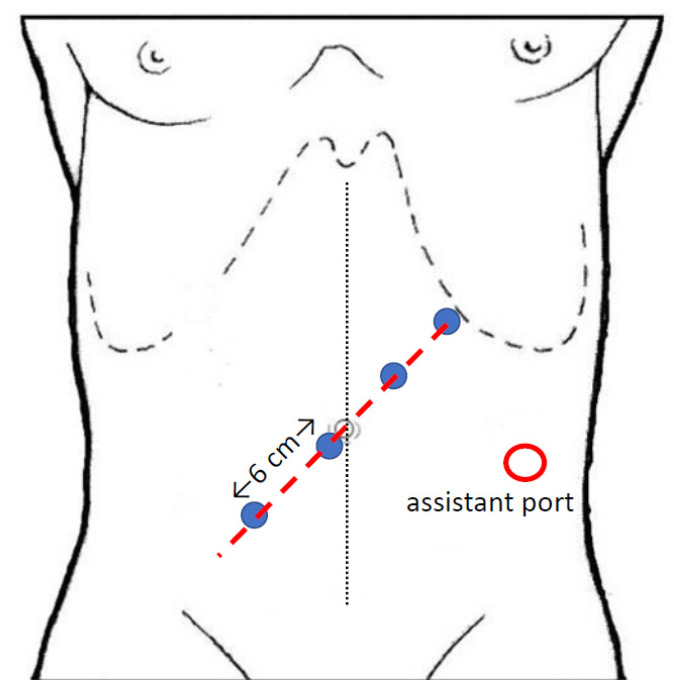
Trocars position for “medial-to-lateral”, “SMV first” and “top-to-down” approach with the da Vinci Xi^®^ system.

In the “medial-to lateral” approach, when using the da Vinci Si^®^ platform, trocars are positioned in the left abdomen with the camera in the left flank (Figure 1a,b); while using the da Vinci Xi^®^ system, all the trocars are along the same line with an oblique costofemoral layout (Figure 2).

With the third robotic arm suspending cranially the transverse mesocolon, the procedure starts with opening of the peritoneum just below the prominence of ileocolic vessels and along the left side of superior mesenteric vein (SMV); the ileocolic artery and vein are then easily identified, dissected and ligated. Subsequently, vascular dissection proceeds cranially with ligature of right colic vessels, middle colic vein and right branch of middle colic artery.

CME is performed once vascular dissection is completed, by sharp separation of posterior mesocolic fascia from retroperitoneum.

This is similar to the “superior mesenteric vein first” approach described by Yang [27] and adopted by other authors [33,34]; the vascular dissection is performed first, exposing the anterior aspect of the SMV by removing the lymphatic tissue covering it, and sequentially ligating the right colic and right branches of middle colic vessels; afterwards the CME is performed by sharp dissection of the ascending and transverse mesocolon from retroperitoneum, exposing the duodenum and the head of the pancreas, and proceeding from medial to lateral until the right colo-parietal area.

**Figure 3:**
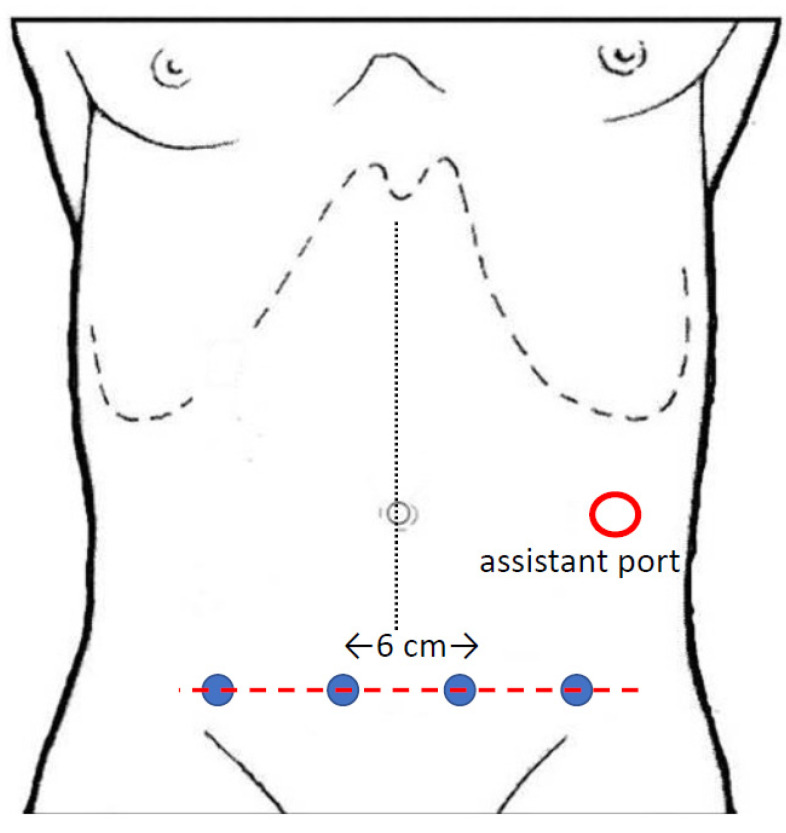
Trocars position for “bottom-to-up” approach with the da Vinci Xi^®^ system.

The “bottom-to-up” approach has been introduced with the da Vinci Xi^®^ platform, which allows the thinner robotic arms to be positioned on the same suprapubic line (Figure 3): with this different vision, frontal to the axis of superior mesenteric vessels, the dissection starts with the incision of the root of mesentery and proceeds cranially developing the retro-mesocolic plane, separating ascending and right mesocolon from the retroperitoneum and joining the ventral aspect of the duodenum and the pancreatic head. Once the is CME completed, vascular dissection is performed, exposing the ventral aspect of SMV and ligating the ileocolic, right colic and right branches of the middle colic vessels.

The “top-to-down” technique has been proposed [26] for the surgical approach to cancer of the distal ascending colon, hepatic flexure or proximal transverse colon: with an oblique offset costofemoral trocars layout (Figure 2), the procedure starts with opening of the gastrocolic ligament and the identification and section of gastroepiploic vessels; the gastro-epiploic vein is then used as a guide to identify and dissect the gastro-colic trunk and, subsequently, the SMV, with removal of the lymphatic and fatty tissue of its anterior aspect. The second phase of the surgical procedure entails a robot redocking with a change of patient position to a 30° Trendelenburg; with the same trocars layout, the vascular dissection is performed and then the CME is realized with a medial-to-lateral direction.

The ileo-colic anastomosis was performed intracorporeally in ten out of the fourteen studies [22,23,24,25,29,31,32,33,34,35] and extracorporeally in two [28,30]; both the techniques have been utilized in two studies [26,27], in which, however, the number of patients receiving intracorporeal or extracorporeal anastomosis is not specified.

## 5. Clinical Outcomes

### 5.1. Operative Results

Conversion rate was reported in all the studies and ranged from 0 to 3.8%; among the four comparative series of robotic versus laparoscopic CME, only Spinoglio [24] reported a significantly higher conversion rate in the laparoscopic than in the robotic group (7% vs. 0).

The mean reported operative time was 236 min; in all the four comparative studies, operative time in the robotic group was significantly higher than in the laparoscopic group.

Intraoperative complications were only reported by Yozgatli [26], who described two cases of minor vascular injuries that were repaired robotically and did not require conversion to open surgery. In the comparative series from Spinoglio [24], a patient in the laparoscopic group had an intraoperative lesion of SMV.

### 5.2. Early Clinical Results

Postoperative complications were detailed in all the studies, with a mean incidence of 22% and no significant differences found in the four comparative studies of robotic versus laparoscopic CME.

The majority of complications were Clavien–Dindo Grade I–II, while median incidence of Clavien–Dindo Grade III–IV complications was 2.8% (range 0–11.5%).

Anastomotic leak rate was very low (0–2%) with the majority of the studies (ten out of fourteen) [22,23,26,27,28,32,33,34] reporting no leaks in the study population.

Early postoperative mortality was declared in only one study [24] and related to a sudden cardiac death.

## 6. Oncologic Outcomes

Adequacy of resection was mainly evaluated by reporting the median number of harvested lymph nodes, which totalled 32 (range 19–41) considering all the studies; in the comparative series published by Ngu [25] and Yozgatli [26], significantly more lymph nodes were retrieved in the robotic group in comparison with the laparoscopic group (41 vs. 31 and 41 vs. 33, respectively), while differences were not significant in the remaining two comparative series.

The length of the specimen was only reported in four studies [22,23,24,31] and its median value was 38.5 cm.

The integrity of the mesocolon was reported in only one study [22].

Only three studies reported on long-term oncologic outcomes: Spinoglio [24], with a median follow up of 60 months, described a 5-year overall survival (OS) rate of 77% vs. 73%, a 5-year cancer specific survival (CSS) of 90% vs. 85% and a 5-year disease-free survival (DFS) of 85% vs. 83%, respectively, in the robotic versus the laparoscopic group; in the specific subgroup of stage III patients, 5-year DFS was 81% versus 68%. Although more noticeable, this, as the other oncologic outcomes, did not differ significantly among the two groups.

In the study from Bae [29], median follow up was 55 months, and OS and DFS were 93% and 81%, respectively.

Siddiqui [34] reported an OS and a DFS of 94%. However, follow up was shorter (3 years).

A summary of clinical and oncologic outcomes of the four different surgical approaches described (“medial to lateral”, “bottom-up”, “top-to-down” and “SMV first”) is depicted in Table 3.

## 7. Discussion

This review underlines the feasibility and safety of the robotic approach to CME with CVL for right colon cancer; in all the reported studies, operative, clinical and oncologic outcomes were good, with a very low rate of conversions to open surgery, a low rate of postoperative serious complications and anastomotic leaks, and satisfactory oncologic results.

Although laparoscopic surgery is considered the gold standard treatment for colon cancer owing to its better short-term outcomes in comparison to open surgery [36,37,38], laparoscopic right colectomy with CME is generally adopted by expert surgeons in high volume Centers [39,40,41].

One of the major concerns regarding the adoption of minimally invasive CME is its intrinsic technical challenge [42]. This is particularly the case when approaching right colectomy. In fact, the extensive dissection of the superior mesenteric vein and artery, to perform an extended lymphadenectomy, and the posterior approach to the transverse mesocolon to expose the second duodenum and the head of pancreas are undoubtedly more complex than the corresponding procedure for left colectomy. In the left colectomy, the dissection plane from the left Toldt’s and Gerota’s fascia is easier to approach, and only two major vessels (the inferior mesenteric artery and vein) have to be isolated and sectioned.

Vascular anatomy of the right colon is more complex [43]; right colonic vessels are not always constant, as the course of ICA after its emergence from SMA (anterior or posterior to SMV). Furthermore, the dissection of Henle’s trunk and the preservation of its pancreatic and gastroepiploic afferents presents an added difficulty.

Surgeons performing a robotic approach to the vascular dissection of a right colectomy with CME are of the opinion that the robotic assistance can play a role in decreasing its technical difficulties; this concept has been widely reported by all the authors of the papers included in this review.

Similarly, some authors have raised concerns of a major risk of vascular injury when CME started to become surgically widespread [44]. This concern was confirmed by preliminary results of an ongoing randomized, controlled, phase 3 superiority trial on laparoscopic right colectomy with CME versus D2 lymphadenectomy [45]. However, it has not been confirmed in our present review of robotic series, as only one author described two cases of minor vascular injury, both of which were successfully repaired and did not require conversion to open surgery. Robotics would, therefore, seem to promise great potential in assuring the feasibility of complex and precise surgical maneuvers.

The operative feasibility of robotic CME is confirmed by the very low incidence of conversions to open surgery (0% in eleven out of the fourteen included studies); in the largest comparative series [24], a significant lower conversion rate in the robotic group is reported.

This is in accordance with evidence from the peer-reviewed published medical literature regarding robotic versus laparoscopic colorectal surgery, where the low conversion rate is accepted as one of the predominant advantages of robotics [46,47]

Finally, the dexterity of the robotic platform with the seven degrees of freedom of surgical instruments has been evoked as decisive in increasing the use of intracorporeal anastomosis (ICA) in comparison to laparoscopic surgery [48,49,50,51]; in all but two studies included in this review [28,30], an ICA was performed and the specimen was extracted through a Pfannenstiel incision. Although level 1 evidence from the literature concerning the advantages of ICA over extracorporeal anastomosis (ECA) after right colectomy is still lacking, [52,53] a recent systematic review and meta-analysis including more than 4400 patients [54] demonstrated that patients receiving an ICA had a significantly lower incidence of conversion to open surgery, total complications, anastomotic leakage, surgical site infection and incisional hernia compared to the ECA group.

If the surgical principles of CME are respected, patients’ oncologic outcomes are likely to improve, as has been shown in previous series, although these are mainly retrospective and non-randomized [4,5,6,7,8,9,10,11,12,13,14].

Therefore, assessment of the adequacy of the surgical resection is mandatory when considering CME surgery; this has been evaluated by number of harvested lymph nodes, length of surgical specimen, distance of tumor from the vessels ligation site, integrity of the mesocolon and plane of surgery achieved. However, all these parameters are not always detailed in the studies reporting on CME, which makes it difficult to obtain definitive and homogeneous results.

Recently, Benz [55] proposed a new classification of surgical specimens of right colectomy with CME, with the aim of standardizing the histopathological evaluation. It takes into account two main parameters: the integrity of the mesocolon and the completeness of tissue removal. The focus, therefore, is on one of the key factors of CME, that is the clearance of the part of the mesocolon covering the anterior surface of SMV between the stumps of ileo-colic and middle colic vessels.

In new trials on CME for right colon cancer, a homogeneous adoption of this new classification is suggested, to standardize and, therefore, compare results.

Among the studies included in this review, only Trastulli [22] mentioned that the quality of mesocolic excision was assessed referring to the West classification of plane of resection, while in all the other studies the only evaluated histopathologic parameters were number of harvested lymph nodes and [22,23,24,31] the length of specimen.

It can, therefore, only be affirmed that the number of lymph nodes number was oncologically appropriate, and that in two of the comparative series, more lymph nodes were retrieved in the robotic group than in the laparoscopic group. However, in the majority of the reported series, principles of CME were declared by authors when describing the surgical technique but could not be verified on surgical specimens.

Regarding oncologic results, understood in terms of OS and DFS, these were very good but only reported in three papers.

The results of the four different approaches to robotic CME described in this review are shown in Table 2. Excluding the “top-to-down” approach reported only in one series [26], clinical and oncologic outcomes of the “medial-to-lateral”, “bottom-to-up” and “SMV-first” approach do not show statistically significant differences.

Nevertheless, the number of harvested lymph nodes is greater in the “bottom-to up” and in the “SMV first” approaches in comparison with the “medial-to-lateral”.

One of the advantages of the “bottom-to-up” approach is the frontal vision of superior mesenteric vascular axis, that can make extensive vascular dissection and D3 lymphadenectomy easier to perform in comparison with the “medial-to-lateral” approach.

Even the “SMV-first” approach is focused on the extensive vascular dissection prior to any other surgical manoeuvre; these technical details, if confirmed by larger scale randomized trials, could in part explain the greater lymph-nodes yield with these two approaches.

In conclusion, this review asserts the feasibility of the robotic approach to CME and CVL for right colectomy; this allows for a low conversion rate, the successful management of any intraoperative complications that might arise, and good clinical outcomes.

Oncologic results should be evaluated, in future trials, by a rigorous assessment of the surgical specimen [55] and by long-term survival rates.

## Figures and Tables

**Table 1:** Characteristics and principal results of the included studies.

Author	Year	Type of Study (Retrosp./Prosp.)	Type of Study (Comp./Non Comp.)	Pat. N	Conversion Rate	Mortality	Leaks	OS	DFS
Trastulli [22]	2013	retrospective	non comparative	20	0	0	0	nd	nd
Petz [23]	2017	retrospective	non comparative	20	0	0	0	nd	nd
Spinoglio [24]	2018	retrospective	comparative	202	0	1%	1%	77% (5 years)	85% (5 years)
Ngu [25]	2018	retrospective	comparative	23	0	nd	nd	nd	nd
Yozgatli [26]	2019	prospective	comparative	96	0	nd	0	nd	nd
Yang [27]	2019	retrospective	non comparative	66	1.5%	0	0	nd	nd
Shulte am Esch [28]	2019	retrospective	non comparative	31	0	0	0	nd	nd
Bae [29]	2019	retrospective	non comparative	43	0	0	2.3%	93.6% (55 months)	81.1% (55 months)
Ramachandra [30]	2020	retrospective	non comparative	52	3.84%	0	1.92%	nd	nd
Petz [31]	2020	retrospective	non comparative	50	0	0	2%	nd	nd
Ceccarelli [32]	2020	retrospective	comparative	55	0	0	0	nd	nd
Larach [33]	2021	retrospective	non comparative	20	0	0	0	nd	nd
Siddiqui [34]	2021	retrospective	non comparative	77	0	0	0	94% (3 years)	94% (3 years)
Bianchi [35]	2021	retrospective	non comparative	161	3.7%	0	0.6%	nd	nd

Pat. n: patients number; retrosp.: retrospective; prosp.: prospective; comp.: comprative; non comp.: non comparativend: not defined; OS: overall survival; DFS: disease-free survival.

**Table 2:** Risk of bias.

Author	Type of Study	NOS	Overall Risk of Biases
Trastulli [22]	non comparative	7 (S4, C1, O2)	low
Petz [23]	non comparative	7 (S4, C1, O2)	low
Spinoglio [24]	comparative	6 (S2, C1, E3)	high
Ngu [25]	comparative	6 (S2, C1, E3)	high
Yozgatli [26]	comparative	6 (S2, C1, E3)	high
Yang [27]	non comparative	7 (S4, C1, O2)	low
Shulte am Esch [28]	non comparative	7 (S4, C1, O2)	low
Bae [29]	non comparative	8 (S4, C1, O3)	low
Ramachandra [30]	non comparative	7 (S4, C1, O2)	low
Petz [31]	non comparative	7 (S4, C1, O2)	low
Ceccarelli [32]	comparative	6 (S2, C1, E3)	high
Larach [33]	non comparative	7 (S4, C1, O2)	low
Siddiqui [34]	non comparative	8 (S4, C1, O3)	low
Bianchi [35]	non comparative	7 (S4, C1, O2)	low

NOS: Newcastle–Ottawa Scale; S: selection; C: comparison; O: outcomes; E: exposure.

**Table 3:** Clinical and oncologic outcomes of robotic CME for right colectomy.

	Surgical Approach				
	Medial-to-Lateral	Bottom-Up	Top-to-Down	SMV First	*p*
Pat. n.	329	328	96	163	
Op. time (median, min)	248	238	286	180	0.07 *
Conversions (median)	0.9%	0.6%	0	0.6%	0.88 °
Postop. compl (median, Dindo III–IV)	3.8%	5.1%	3%	1.8%	0.18 °
Harvested LN (median)	24	34	41	33	0.15 *

*: one way ANOVA test; °: Chi-square test; Pat.: patients; SMV: superior mesenteric vein; Op.: operative; min: minutes; Postop.: postoperative; compl.: complications; LN: lymph nodes.

## Data Availability

All data were retrieved by PubMed search.
